# Osteoimmunology: The correlation between osteoclasts and the Th17/Treg balance in osteoporosis

**DOI:** 10.1111/jcmm.17399

**Published:** 2022-05-28

**Authors:** Fei Huang, Puiian Wong, Jinglan Li, Zheng Lv, Liangliang Xu, Genfu Zhu, Mincong He, Yiwen Luo

**Affiliations:** ^1^ Department of Traumatology The Third Affiliated Hospital of Guangzhou University of Chinese Medicine Guangzhou China; ^2^ Department of Orthopedics The First Affiliated Hospital of Guangzhou University of Chinese Medicine Guangzhou China; ^3^ Laboratory of Orthopaedics & Traumatology, Lingnan Medical Research Center Guangzhou University of Chinese Medicine Guangzhou China; ^4^ Institute of Osteoporosis The Third Affiliated Hospital of Guangzhou University of Chinese Medicine Guangzhou China; ^5^ Guangdong research institute for Orthopedics & Traumatology of Chinese Medicine The Third Affiliated Hospital of Guangzhou University of Chinese Medicine Guangzhou China

**Keywords:** osteoimmunology, Th17/Treg, RANKL, osteoclasts, osteoporosis

## Abstract

Osteoporosis is a bone disease that is caused by disorder of the skeletal microenvironment, and it characterized by a high disability rate and the occurrence of low energy fractures. Studies on osteoporosis and related treatment options have always been hot spots in the field of bone biology. In the past, the understanding of osteoporosis has been rather limited; research has only shown that osteoporosis involves the imbalance of bone resorption and bone formation, and recent studies have not provided cutting‐edge theories of the basic understanding of osteoporosis. Recent studies have shown crosstalk between bone and immune responses. RANKL, an essential factor for osteoclasts (OCs), is associated with the immune system. T helper (Th17)/regulatory T (Treg) cells are two different kinds of T cells that can self‐interact and regulate the differentiation and formation of OCs. Therefore, understanding the correlation between the skeletal and immune systems and further revealing the roles and the cooperation between RANKL and the Th17/Treg balance will help to provide new insights for the treatment of osteoporosis.

## 1 INTRODUCTION

Osteoporosis is a systemic metabolic bone disease characterized by deterioration of bone microstructure and decreases in bone density.^1^ Osteoporosis can significantly weaken the bone structure and cause fragile fractures. According to traditional opinions, osteoporosis is the result of many causes, including metabolic disorders, endocrine disorders, glucocorticoid abuse and oestrogen deficiency.^2^ However, in recent years, studies have found that the immune system shares many regulatory factors with the skeletal system. In 2020, Zhang et al.^3^ reviewed the regulatory roles of T lymphocytes (T cells) in osteoporosis. However, recent studies of T cells and osteoporosis have included additional T‐cell subsets, namely, T helper 17 (Th17) and regulatory T (Treg) subsets and osteoclasts (OCs). For example, receptor activator of nuclear factor‐κ B ligand (RANKL), a factor that is necessary for OC differentiation, is released in its soluble form by T helper 17 (Th17) cells in the immune system.^4^ The correlation between OCs and the Th17/Treg balance in osteoporosis has attracted great interest and attention.

In this review, the correlation between OCs and Th17 cells as well as Treg cells is introduced. In addition, we present the impact of the balance between Treg cells and Th17 cells on OCs. Moreover, we summarize the relevant factors that affect the Th17/Treg cell balance to provide new ideas for the treatment of osteoporosis.

## 2 THE CORRELATION BETWEEN THE IMMUNE SYSTEM AND OSTEOPOROSIS

The close relationship between the immune system and skeletal system was previously recognized.^5^ There has been growing evidence that the immune and skeletal systems share many regulatory molecules, including cytokines, receptors, signalling molecules and transcription factors. In 2000, Arron and Choi introduced the relation between bone resorption and T cells and further proposed the concept of ‘bone immunology’ to emphasize the regulatory role of T cells in OC formation in autoimmune arthritis.^6^ Takayanagi et al.^5^, ^6^ indicated that Th17 cells produce IL17 to promote the activation of bone resorption cells by upregulating the expression of NF‐κB. This finding highlights the dynamic relationship between bones and the immune system. Due to the crosstalk and coupling of the bone‐immune system, recent studies have discovered a new mechanism underlying the pathogenesis of osteoporosis and have started to treat osteoporosis by targeting bone immunity. This perspective provides additional opportunities for the treatment and research of osteoporosis.^7^, ^8^

Bone marrow is one of the main sites of haematopoiesis and immune system development. One of the main functions of the immune system is providing the first response to infection and injury to limit the spread of infection and activate immune cells to promote tissue repair.^9^ With further research, it has been shown that bone cells also regulate immune cells. The specific genetic loss of the G protein subunit in osteoblasts leads to a decrease in B‐cell precursor numbers in bone.^10^ A study confirmed that osteoblasts were a part of a specific B‐cell niche in bone marrow.^11^ OC progenitor cells can differentiate into dendritic cells and macrophages.^9^ which indicates that OCs are closely related to the immune system. Murine dendritic cells promote the formation of OCs in vivo.^12^ In the bone microenvironment, repair and regeneration provide a scaffold for maintaining bone physiology. Continuous bone resorption is required for bone homeostasis and remodelling constantly occurs to adapt to loading and to replace damaged bone tissue. Tissue repair and the internal balance of bone require the innate immune response.^13^

## 3 THE RELATIONSHIP BETWEEN TH17/TREG CELLS AND OSTEOPOROSIS

### 3.1 The relationship between OCs and T cells

Helper T cells and regulatory T cells, two subsets of T cells, have been studied in recent years.^14^ Their mutual transformation, mutual antagonism and mutual influence, as well as the underlying mechanisms are worth exploring. The regulation of OC formation and bone resorption is achieved by the secretion of RANKL by these T‐cell subsets. This recently identified interaction between the skeletal system and immune system is changing the current understanding of the mechanism underlying osteoporosis.^4^, ^15^ At present, there are substantial limitations in the treatment of osteoporosis with anti‐absorptive drugs and bone‐promoting drugs. According to this newly discovered mechanism, some new methods that regulate the balance of T cells in the immune system may allow the regulation of bone resorption and bone formation.

Osteoclasts are the main mediators of skeletal diseases, especially osteoporosis. In addition to the roles related to bone resorption in bone diseases, OCs also function in adaptive immunity.^16^ OC‐related signalling pathways and the receptor activator of nuclear factor‐κB (RANK)/RANKL/osteoprotegerin (OPG) pathway functionally control both OCs and peripheral T cells. This reflects the close similarities between OC and T‐cell biology.^17^ In recent years, there have been emerging ideas that cells of the OC lineage exert direct immunosuppressive effects on adaptive immunity. OCs share a common cell source with macrophages and dendritic cells. It has been observed that OCs present antigens to T cells.^18^

### 3.2 Production of RANKL in the immune system

**FIGURE 1:**
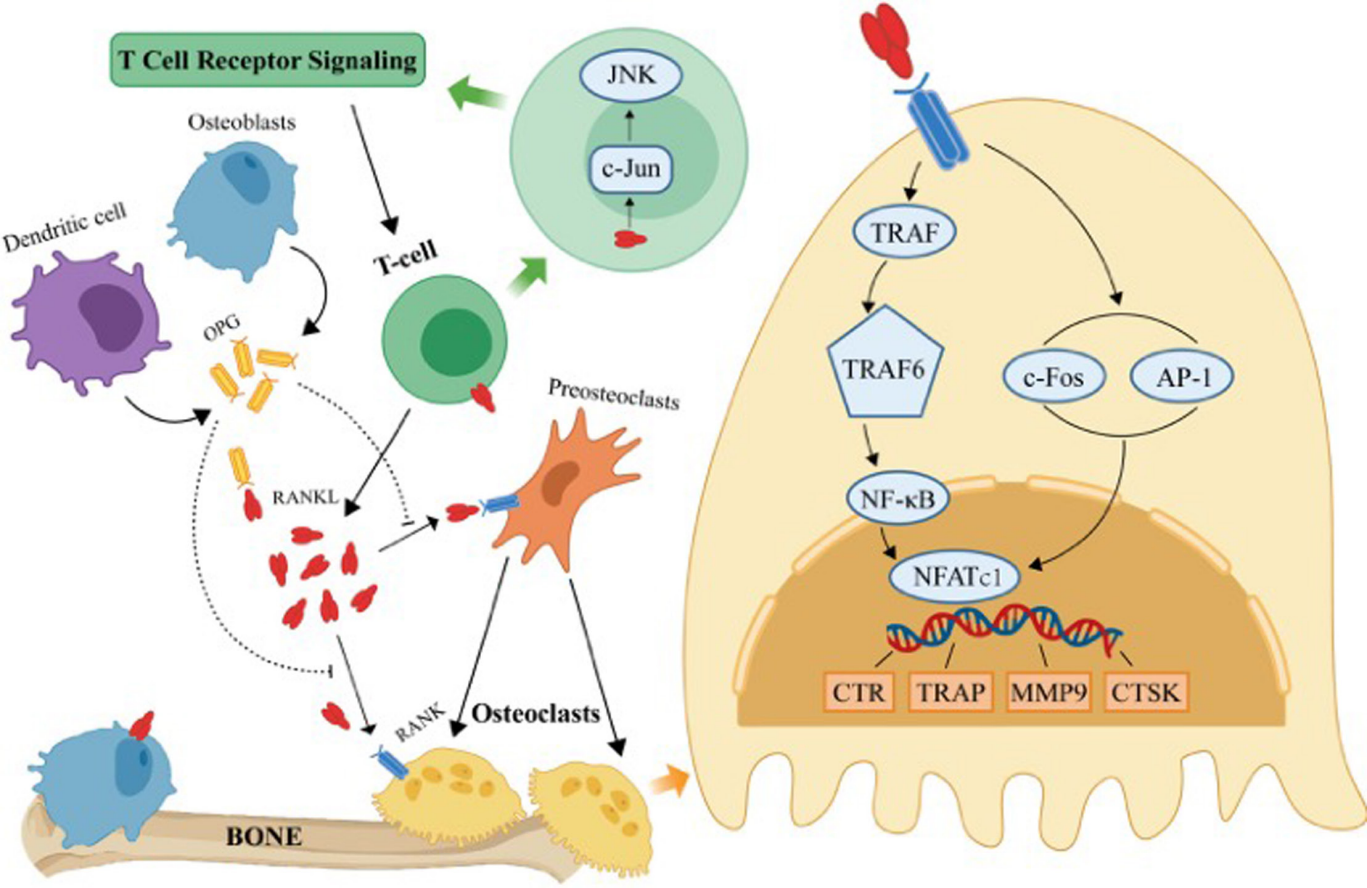
RANK/RANKL/OPG pathway map. Activated T cells induce the activation of the JNK pathway by activating c‐Jun, resulting in signal transduction. T cells secrete a large amount of RANKL, which binds to its receptor rank, recruits TRAF, activates the signalling pathway downstream of TR6AF; alternative, RANKL promotes the production of OCs by inducing C‐fos and AP‐1 activation to promote the expression of NFATC1, resulting in an increase in OC production. OPG secreted by osteoblasts and dendritic cells acts as bait receptors for RANKL, which competitively inhibits the binding of RANKL to RANK, thereby inhibiting OC differentiation

In 1972, Choi and his colleagues discovered a new member of the tumour necrosis factor (TNF) cytokine family, namely, TRANCE (TNF‐related activation‐induced cytokine, also named RANKL). The tumour necrosis family is derived from dendritic cells, which are a type of haematopoietic cell that can capture, process and present antigens to T cells and participate in immune surveillance. TRANCE is a transcription factor whose expression is upregulated by stimulation and calcineurin of the T‐cell receptor. RANKL, which exists in a recombinant soluble form, is produced by T cells, induces activation of the C‐Jun amino terminal kinase (JNK) in T cells, and participates in T‐cell signal transduction. RANKL plays a crucial role in regulating the T‐cell immune response.^19^ RANKL can activate dendritic cells to stimulate the proliferation of T cells, and it can improve the survival rate of RANKL^+^ T cells via interleukin‐4 and transforming growth factor. In 1999, Penninger et al.^20^ found that mice with a disrupted RANKL gene suffer from osteoporosis and tooth eruption defects. In these mice, although the structure of the spleen and development of dendritic cells were normal, the differentiation and development of early T cells were dysfunctional. Therefore, it is thought that this gene can regulate the development of the lymphatic system, and it has been proven to be an important factor in OC differentiation. The latest results show that the same molecule that regulates OCs can also affect the development of cells in the immune system, such as thymocytes and B cells. This illustrates the close relationship between RANKL and the immune system.^21^, ^22^ (Figure 1).

### 3.3 RANKL and Th17/Treg

Th17 cells regulate the RANKL/RANK/OPG pathway in direct and indirect ways. Osteoclast precursor cells directly express RANKL, and when it binds to RANK, these cells are promoted to become osteoclasts. Chen et al.^5^ showed that bone loss is related to the ratio of bone remodelling and RANKL/OPG expression, and the expression of RANKL increases as the number of Th17 cells increases. In an indirect manner, IL‐17 secreted by Th17 cells can stimulate osteoclastogenesis‐supporting cells, such as synovial fibroblasts and osteoblasts, to express RANKL and increase bone resorption. Moreover, Th17 cells can induce the production of inflammatory factors, such as TNF‐α, IL‐1 and IL‐6. Accompanying inflammatory infiltration, the increased expression of NF‐κB can further promote the expression of RANKL. Sato et al.^23^ revealed that IL‐17 is critical for bone destruction in a rheumatoid arthritis mouse model. In an ovariectomy rat model, the level of IL‐17 was shown to be increased, and anti‐IL‐17 treatment was reported to prevent the bone destruction caused by oestrogen deficiency.^24^

Treg cells have become a hotspot in osteoimmunology since they were first reported in 1995. Unlike Th17 cells, which function in immunosuppression, Treg cells suppress the levels of RANKL and M‐CSF, which are both necessary for osteoclast formation, to restore bone mass.^25^ The inhibition of RANKL in Tregs resulted in attenuated migration and decreased production of the cytokines IL‐10 and TGF‐β. Transfer of CD4^+^Foxp3^+^ Tregs into mice that received anti‐RANKL treatment restored the ability of these cells to regulate immune responses, reduced inflammatory responses in skin lesions, restored normal T‐cell responses in vivo and in vitro, and prevented the recurrence of skin lesions after the cessation of anti‐RANKL treatment. Therefore, RANKL inhibitors not only effectively inhibit bone loss but also promote host inflammation by interfering with Treg activity. This suggests that this classic OC mediator exerts an immunomodulatory effect. The absence of RANKL also inhibits Tregs and decreases Treg function. After anti‐RANKL drug withdrawal, a slight increase in the number of Tregs was observed, indicating that the changes in immunoregulation may be reversible and last longer than the rapid reversible effects observed in bones. Therefore, the effects of RANKL on Tregs represent a feedback mechanism underlying immune regulation.^4^

**FIGURE 2:**
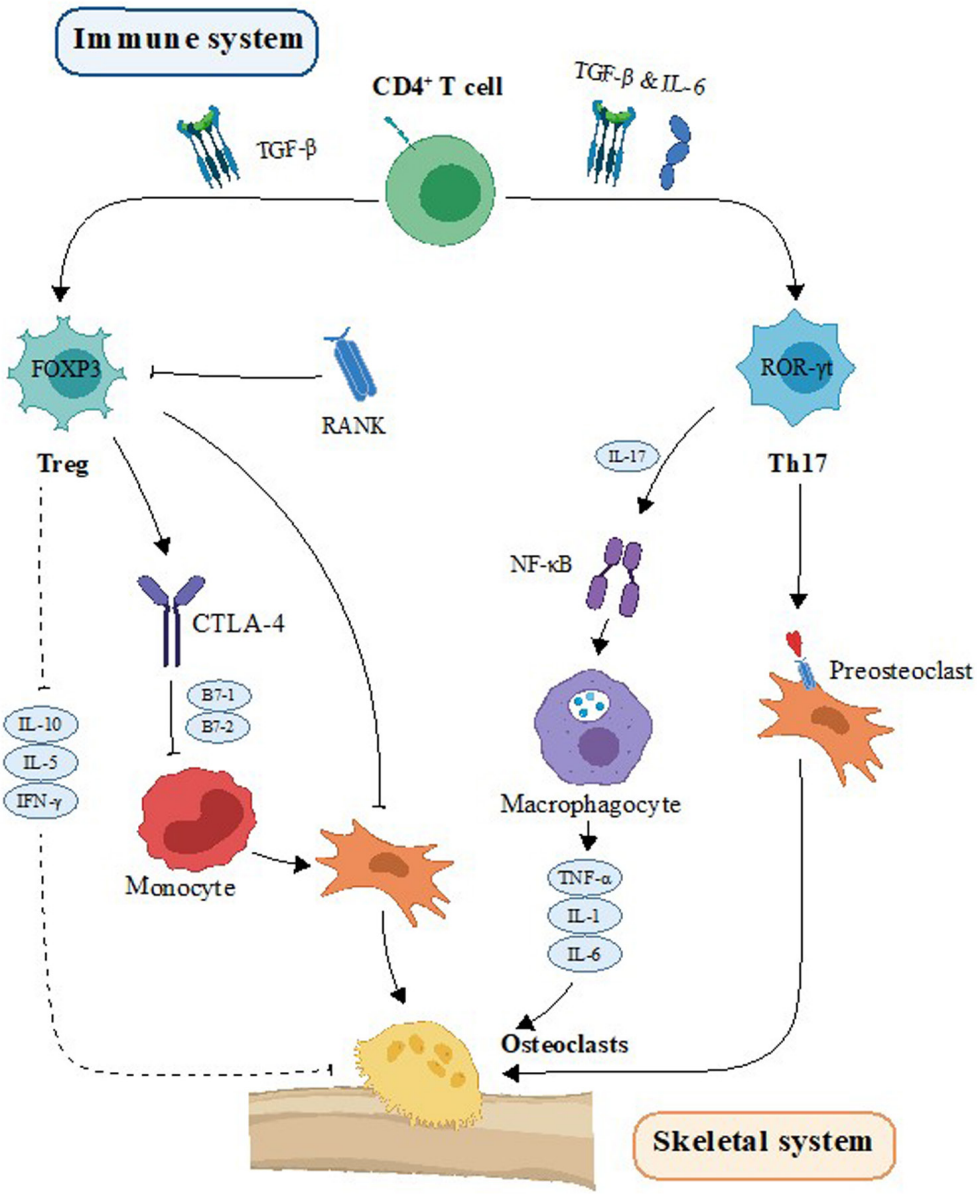
The immune system and bone system show that primitive CD4+ T cells need TGF‐β to differentiate into different T cell subsets. These cells differentiate into Th17 cells in the presence of IL‐6 and into Treg cells in the presence of TGF‐β alone. Th17 cells mainly express ROR‐γt to promote the formation of OCs through the interaction of RANKL and RANK, which leads to an increase in OC numbers. IL‐17, another cytokine produced by Th17 cells, can increase the number of macrophages by increasing the expression of NF‐κB, leading to an increase in the levels of various proinflammatory factors and resulting in the maturation of OCs. There are three mechanisms by which Treg cells inhibit OC formation: (1) Inhibition of OC formation through direct contact between cells; (2) inhibition of OC formation by the secretion of anti‐inflammatory factors. (3) inhibition of the generation of monocytes by CTLA‐4, which is highly expressed in TREGs, resulting in a decrease in OC production

Treg cells can inhibit OC formation mainly through direct contact and cytokine‐dependent mechanisms. CTLA‐4 (cytotoxic T lymphocyte‐associated antigen 4), which is highly expressed in Treg cells, binds to the B7‐1 costimulatory factor and B7‐2 costimulatory molecule on monocytes. CTLA‐4 inhibits OC formation in a dose‐dependent manner by binding to OC precursors. The neutralization of CTLA‐4 completely restored the inhibitory effect of Treg cells on OCs.^26^ IL‐10, a Treg cell‐derived cytokine, can not only suppress the proliferation of T cells but also downregulate the levels of RANKL and M‐CSF to inhibit the maturation of osteoclasts.^27^ In addition to IL‐10, IL‐35 secreted by Treg cells can reduce the production of IL‐17 and inhibit the osteoclastogenesis factor from IL‐17.^28^ These main factors together reshape the relationship between bone and immunity, further enhancing the relationship between inflammation and osteoporosis (Figure 2).

#### 3.3.1 The relationship among OCs, Th17/Treg cells and inflammation in osteoporosis

Postmenopausal oestrogen deficiency triggers proinflammatory cytokine production, leading to osteoporosis. There is promising evidence suggesting that tumour necrosis factor‐α (TNF‐α), IL‐6, IL‐11, IL‐15 and IL‐17 are involved in bone resorption, resulting in an increase in OC numbers.^29^, ^30^ Animal studies have indicated that proinflammatory cytokines are mediators of bone resorption in postmenopausal women.^31^ The role of T cells in skeletal ecology is rarely discussed. Many studies have shown that regulating the T‐cell balance can reduce OC formation, and thus, slow bone resorption.^32^, ^33^, ^34^, ^35^

Chronic inflammatory diseases, such as rheumatic arthritis (RA), gout, psoriatic disease and osteoarthritis, are often accompanied by osteoporosis or bone resorption.^36^ Increased levels of inflammatory cytokines, including RANKL, lead to enhanced osteoclast activity and bone damage in RA patients.^37^

## 4 TREATMENTS OF OSTEOPOROSIS

### 4.1 Current treatments for osteoporosis

The treatments for osteoporosis are usually divided into treatments that promote bone formation and treatments that prevent bone resorption. Oestrogen replacement therapy is commonly used for the prevention and treatment of osteoporosis in postmenopausal females.^1^ Bisphosphonate is a drug that prevents bone resorption, and it changes the morphology of OCs, interfering with bone resorption by binding to bone matrix and inhibiting the production of osteoblast‐derived cytokines, such as IL‐6 and TNF.^38^, ^39^ Preliminary studies have shown that bisphosphonates can prevent OC repair by inhibiting cytokine production by osteoblasts. Denosumab is an anti‐RANKL monoclonal antibody that blocks the function of RANKL in the process of bone loss and achieves a therapeutic effect.^40^ It is clinically proven that Denosumab can increase bone mineral density (BMD) and reduce the incidence of vertebral and hip fractures compared with placebo. However, the effects of denosumab on the immune system are poorly understood. For example, initiation of denosumab treatment can cause a reduction in Treg cell numbers, and it might strengthen antitumor immunity in cancer patients.

### 4.2 Potential treatment for osteoporosis related to Th17/Treg dysregulation

#### 4.2.1 IL‐17

IL‐17, a powerful factor that drives OC production, has been shown to exacerbate bone destruction and bone loss. IL‐17 can directly bind to RANKL and promote OC formation. When the IL‐17 signalling pathway is blocked, a positive improvement in autoimmune diseases is observed. Additionally, it was discovered that IL‐17R with a functional domain called ‘SEFIR’ is essential for IL‐17 signal transduction because the absence of this region impairs IL‐17R signal transduction.

IL‐17 promotes the secretion of prostaglandin E2, a promoter of OC formation, by osteoblasts.^41^ Although further clinical trials are needed to determine the efficacy, safety and tolerance of cytokine inhibitors, these strategies may provide novel treatment strategies for osteoporosis.

#### 4.2.2 Chinese medicine immunotherapy‐monomer

Traditional Chinese medicine has unique advantages for regulating the interaction between the immune system and osteoporosis. New treatments based on syndrome differentiation provide new opportunities for the immunotherapy of osteoporosis. Traditional Chinese medicine compound prescriptions and monomers provide a basis for syndrome differentiation and ideas for the immunotherapy of osteoporosis.

Sinomenine is widely used in the treatment of inflammatory diseases. It can ameliorate rat arthritis by regulating T cells and inhibiting the formation of OCs.^42^, ^43^ Studies have shown that *Tripterygium wilfordii* is an herb with strong anti‐inflammatory properties. It plays an immunomodulatory role in bone marrow stromal cells by decreasing the ratio of RANKL/OPG, thus inhibiting the differentiation of OCs. Naringin has been shown to suppress the expression of inflammatory factors and inflammation caused by the overexpression of NF‐κb.^44^ *Eucommia ulmoides* is a classic medicine that is used to ‘tonify the kidney’ in traditional Chinese medicine. From the perspective of traditional Chinese medicine, the cause of osteoporosis is related to kidney deficiency. This pathogenesis is consistent with the pharmacological action of *Eucommia ulmoides*. Both modern medicine and traditional medicine have supported the theoretical basis of the use of *Eucommia ulmoides* and its monomers for the treatment of osteoporosis.^45^, ^46^

## 5 CONCLUSION

In recent years, the definition of osteoimmunology has resulted in new opinions about osteoporosis. The role of inflammation has been closely associated with bone loss and osteoclastogenesis. Bone loss is also associated with the immune system. It may be determined that autoimmunity triggers inflammation, further resulting in bone loss and leading to osteoporosis. Abnormal immune responses cause autoimmune diseases, which leads to an increase in proinflammatory factor levels. The enhancement of bone resorption promotes the occurrence and development of osteoporosis. It has been shown that osteoporosis results from combinations of dysfunction, including the dysregulation of the immune system and the Th17/Treg cell balance. In this article, we have reviewed the treatment strategies that target immune regulation. In the future, more studies are needed to explore the mechanism underlying the bone‐immune interaction in osteoporosis and to explore additional new drugs that are clinical effective and safe.

## AUTHOR CONTRIBUTIONS

**Fei Huang:** Writing – review and editing (equal). **Puiian Wong:** Writing – review and editing (equal). **Jinglan Li:** Writing – original draft (equal). **Zheng Lv:** Writing – original draft (equal). **Genfu Zhu:** Supervision (equal). **Liangliang Xu:** Project administration (equal). **Mincong He:** Methodology (equal). **Yiwen Luo:** Project administration (equal).

## CONFLICT OF INTEREST

The authors have no conflicts of interest to disclose in relation to this article.

## Data Availability

Not available.
